# The Elements of Materia Medica and Therapeutics

**Published:** 1843-04-29

**Authors:** 


### BIBLIOGRAPHICAL NOTICES.

*The Elements of Materia Medica and Therapeutics.* By JONATHAN PEREIRA, M. D., F. R. S. and L. S., &c. &c. With Numerous Illustrations. From the Second London Edition, Enlarged and Improved. With Notes and Additions. By JOSEPH CARSON, M. D., &c. In Two Volumes. 8vo. pp. 714–852. Philadelphia: Lea & Blanchard.

The work of Dr. PEREIRA has been for several years familiar to the student of Materia Medica in this country. Its present republication throws it open to the profession generally. It is, beyond all doubt, the most complete work of the kind in our language. The amount of learning and research exhibited is almost incredible, and, along with these, there is much sound personal knowledge, with professional tact and good judgment. The work is at once copious and concise; there is nothing in connection with the subject which, on reference to its pages, we cannot find, and yet all this is done in a compact, happy manner. "Replete with erudition, and at the same time most satisfactory with respect to references, it is admirably suited to the wants of the advanced student and practitioner; whilst from the distinctness of the facts, their methodical arrangement, and the clear philosophical explanations connected with them, it meets the wants of the student who is in search of the first lessons in the science. It may, therefore, with equal benefit, be employed as a work of reference, or as an elementary text book, in which two-fold character it occupies an unusual position."—(*American Editor's Preface.*)

To adapt it to our meridian, the nomenclature of the last edition of the United States Pharmacopœia has been introduced. This has been done by "inserting the name of each article adopted by that standard, in connexion with the authorities uniformly cited by the author, or by expressing a correspondence of name with one or more of them by the symbols (U. S.) in union with similar symbols, used by him to indicate the authority." Where the formulæ of our national standard are new, or differ from those in the text, they have been presented in detail. Histories of our more important indigenous medicines have been introduced. The manner in which the American editor has discharged his delicate and onerous duties is highly creditable to him, and show how amply qualified he was, in all respects, for the task. He has shown at once knowledge and judgment.

A Tabular Historical View of the History and Literature of the Materia Medica precedes Part First, which embraces General Therapeutics, a complete and excellent treatise in itself. Part Second treats of Special Pharmacology. The articles are arranged in Natural Historical Order. Under the Inorganized Kingdom the Non-metallic and Metallic Substances are treated. The Organized Kingdom is divided into two sub-kingdoms—the Vegetable and Animal.

The work is illustrated with two hundred and seventy-nine wood cuts.

